# Visual motion processing deficits in infants with the fragile X premutation

**DOI:** 10.1186/1866-1955-6-29

**Published:** 2014-07-30

**Authors:** Pamela K Gallego, Jessica L Burris, Susan M Rivera

**Affiliations:** 1 Department of Psychology, University of California Davis, Davis, CA 95618, USA; 2 Center for Mind and Brain, University of California Davis, 202 Cousteau Place, Suite 250, Davis, CA 95618, USA; 3 M.I.N.D. Institute, University of California Medical Center, Sacramento, CA 95817, USA

**Keywords:** Premutation, Visual processing deficits, Fragile X syndrome, Contrast detection

## Abstract

**Background:**

Fragile X syndrome (FXS) results from a trinucleotide repeat expansion (full mutation >200 cytosine-guanine-guanine (CGG) repeats) in the *FMR1* gene, leading to a reduction or absence of the gene’s protein product, fragile X mental retardation protein (FMRP), ultimately causing cognitive and behavioral impairments that are characteristic of the syndrome. In our previous work with infants and toddlers with FXS, we have been able to describe much about their cognitive and visual processing abilities. In light of recent work on the mild cognitive deficits and functional and structural brain differences that are present in adults with the fragile X (FX) premutation, in the present study we examined whether some of the low-level visual processing deficits we have observed in infants with FXS would also be present in infants with the FX premutation (55–200 CGG repeats).

**Methods:**

We chose a contrast detection task using second-order motion stimuli on which infants with FXS previously showed significantly increased detection thresholds (Vision Res 48:1471–1478, 2008). Critically, we also included a developmental delay comparison group of infants with Down syndrome (DS), who were matched to infants with FXS on both chronological and mental age, to speak to the question of whether this second-order motion processing deficit is a FX-specific phenomenon.

**Results:**

As reported previously, infants with the FX full mutation showed motion contrast detection threshold levels that were significantly higher than age-matched typically developing control infants. Strikingly, the motion detection contrast levels of FX premutation infants were also significantly higher than typically developing (TD) infants and not significantly different from the group of infants with FXS or with DS.

**Conclusions:**

These results, which are in keeping with a growing body of evidence on the mild cognitive and perceptual processing deficits and functional and structural brain differences that are present in adults and older children with the FX premutation, underscore the pressing need to study and describe the processing capabilities of infants and toddlers with the FX premutation.

## Background

Fragile X syndrome (FXS) is the most common inherited cause of mental disability, which results from a reduction or absence of the fragile X mental retardation protein (FMRP), a gene product known to play an essential role in brain structure and function [1,2]. This condition emerges when the repeat expansion of the trinucleotide cytosine-guanine-guanine (CGG) in the 5′ untranslated region of the *FMR1* gene, located in the X chromosome, is above 200 repeats. When this occurs, the *FMR1* gene is typically fully methylated, which prevents the transcription and the translation of the gene, consequently disrupting the production of FMRP and leading to cascading cognitive and behavioral impairment, including mild to severe intellectual disability, social anxiety, math and spatial reasoning problems, and relatively high co-morbidity with autism (30% of all FXS cases) [3,4]. The number of individuals with FXS (full mutation >200 CGG) ranges approximately between 1 per 2,500 in females to 1 per 4,000 in males [5].

Individuals with CGG repeat expansions of 55–200 are considered to be premutation carriers of FXS, a condition more commonly found in the general population, affecting approximately 1 in 130–250 women and 1 in 250–810 males [5]. Individuals in the premutation range typically have normal intellectual functioning, but may have elevated *FMR1* mRNA, in some cases three to eight times the normal levels [6]. This elevated mRNA is thought to produce RNA toxicity, which has been associated with mild deficits in working memory [7], memory encoding [8], memory recall [9], enumeration [10], and increased psychiatric symptoms including obsessive-compulsive symptoms and psychoticism [11]. In addition, male premutation carriers, especially, are at risk of developing the late-onset neurodegenerative disorder known as fragile X-associated tremor/ataxia syndrome (FXTAS) [7,12].

There is now ample evidence that young children with FXS have significant visual-spatial impairments. For example, studies of infants and toddlers with FXS have documented impairments in processing texture-defined (second order) motion stimuli [13], temporal flicker [14], perceiving the ordinality of sequences of numerical displays [15], and the ability to maintain the identity of dynamic object information during occlusion [16]. Impaired performance has also been demonstrated on tasks requiring visual-motor responses [17,18] as well as inhibitory control [19] and numerical reasoning [20,21]. One purported cause of the visual-spatial and numerical deficits seen in FXS is disruption of the so-called dorsal stream (the occipito-parietal visual pathway, projecting to the posterior parietal cortex, which processes information involved in guiding actions, including spatial location and motion) with relative sparing of the *ventral stream* (the occipito-temporal visual pathway, projecting to the inferior-temporal cortex, which processes object features such as form and color) [22,23]. Because of its relatively protracted time course of development [24], the dorsal stream is thought to be particularly vulnerable to atypical development in a number of disorders, including in FXS [16,25].

Compared to young children with the fragile X full mutation (FXS), very little is understood about the cognitive and visual processing abilities in young children with the fragile X (FX) premutation. Recent work on the mild cognitive deficits and functional and structural brain differences that are present in adults with the FX premutation [7,8,10,11,26] and particularly studies which have documented deficits in visuospatial [27,28] and contrast sensitivity [29] in adult premutation carriers led us in the present study to examine whether one of the low-level visual processing deficits that has been observed in infants with FXS is also present in infants with the FX premutation. To study this, we chose a contrast detection task using second-order motion stimuli on which infants with FXS demonstrated significantly increased detection thresholds [13]. We hypothesized that infants and toddlers with the premutation would perform similarly to infants and toddlers with the full mutation, i.e., the threshold necessary for detection of visual stimuli would be higher than typically developing mental and chronological age-matched controls and would not be significantly different from participants with the full mutation. We also included a comparison group of infants with Down syndrome, who are matched with the FX full mutation group on both mental and chronological age, allowing us to examine whether deficits seen in second-order motion processing are specific to the FX-specific spectrum.

## Methods

### Participants

Four groups of participants were enrolled in this study: 16 typically developing infants (7 male and 9 female, mean age 13.17 months), 12 premutation carrier infants (8 males and 4 females, mean age 17.56 months), 24 infants with FXS (19 male and 5 female, mean age 29.24 months), and 15 infants with Down syndrome (5 males and 10 females, mean age 26.27 months). A one-way ANOVA confirmed that the groups significantly differed in their chronological age (*F*(3, 63) = 6.67, *p* = 0.001). Simple effect analyses revealed that there was no significant difference in chronological age (in months) between typically developing (TD) infants (*M* = 13.17; SD = 7.91) and infants with the FX premutation (*M* = 17.56; SD = 12.57; *t*[26] = 1.13, *p* = 0.27) nor between infants with Down syndrome (DS) (*M* = 26.27; SD = 11.95) and FXS (*M* = 29.24; SD = 14.35; *t*[30] = 0.668, *p* = 0.51). By contrast, both the DS and FXS were significantly chronologically older than TD infants (*t*[31] = 4.07, *p* = 0.002; *t*[29] = 3.62, *p* = 0.001, respectively.) For infants with the FX premutation, repeat sizes ranged from 55 to 181, with a mean length of 94. For infants with FXS, CGG repeat sizes ranged from 210 to 702, with a mean length of 466.

Participants with FXS were recruited and clinically evaluated at the UC Davis MIND Institute. Four participants with the FX premutation were seen as patients at the UC Davis M.I.N.D. Institute Fragile X Research and Treatment Center (FXRTC), while eight were recruited through a newborn screening project in which parents in the general population could consent to screen their newborn infants for metabolic abnormalities and other preexisting conditions [32]. This recruitment combination allows us to have a sample of premutation infants that is more representative of the population because parents who enroll in the newborn screening program are unaware of their child’s preexisting condition and therefore do not present the ascertainment bias that may occur in participants who come to the FXRTC clinic seeking resources for their child. Participants with DS were recruited from the community by attending outreach events. Typically developing infants were recruited through letters to families, fliers, and word of mouth.

Participants were developmentally age-matched using the Mullen Scales of Early Learning [33], a standardized developmental assessment used for children 3–60 months consisting of 5 subscales: gross motor, fine motor, visual reception, expressive language, and receptive language. The mental age of each participant was calculated by averaging across the four different domains (VR, FM, RL, and EL) and converting that average to age in months and days. The gross motor subscale was omitted from the mental age calculation as the scores become less valid in children above the age of 33 months [33]. The mean mental age was 13.22 months for typically developing participants, 15.10 months for premutation carriers, 18.01 months for participants with FXS, and 14.13 for participants with DS. A one-way ANOVA confirmed that mental age did not differ significantly between the four groups (*F*(3,63) = 0.850, *p* = 0.472). Table 1 depicts the averaged ELC score across the four groups. A one-way ANOVA confirmed that the ELC scores, as expected, differed significantly between the four groups (*F*(3,63) = 29.67, *p* = 0.000). Post hoc analyses show that there is no significant difference between the participants with the premutation and typically developing group when using Bonferroni-corrected values (*p* = 0.094), suggesting that these two groups are performing at an overall comparable cognitive level.

**Table 1:** Mean scores of the early learning composite scores of the Mullen across groups

**Group**	**Mean ELC score**
TYP	103.33
PRE	87.73
DS	54.93
FXS	65.61

### Apparatus and stimuli

A Tobii 1750 binocular eye tracker monitor (Tobii Technology, Danderyd Sweden, http://www.tobii.com) was used to present the visual stimuli. This eye tracking system consists of a high-resolution camera that records the eye position, embedded in a 17-in. monitor (1,280 by 1,024 pixel resolution, 50-Hz refresh rate) with infrared light-emitting diodes that illuminate the cornea, capturing and tracking eye movements that are then run through proprietary algorithms that calculate changes in eye position. The visual angle subtended by the display was 31.63° by 25.36° region on the screen when viewed from a distance of 60 cm. The data are captured at a frame rate of 50 Hz and sent to Tobii Studio (version 2.0.8) to be overlaid on the stimuli. The stimuli used for this study was generated using The Vision Shell PPC program, controlled by an Apple G4 Power Macintosh with OS9 (Apple, Cupertino, CA, USA). Please see Farzin et al. [13] for a detailed description of the second-order (texture-defined) motion (4 Hz) sine wave gratings stimuli used in this study.

### Procedure

The Institutional Review Board at the University of California, Davis, approved the experimental protocol, and an informed consent was obtained from the parents of all infants. The infants were tested while seated on a caregiver's lap and were positioned so that their face was approximately 60–70 cm in front of the eye tracker. In order to attract the participants' attention to the screen, room lights were dimmed and an attention-getter video was displayed on the screen. During this time, an experimenter monitored the participant's eye position using a real-time track-status monitor. If the participant's eyes were not found, adjustments were made (repositioning the participant or angling the monitor to adjust for the participant's height) until a track status was obtained on both eyes.

Once the participant's eyes were detected by the eye tracker, a five-point calibration routine was executed in Tobii Studio. If all points were acquired, the calibration was saved and the stimulus presentation would begin. If the calibration was not successful (i.e., not all five points were acquired), another calibration was attempted. The minimum criterion required to proceed with the task was a successful calibration of the center point for each eye. This ensured that the gaze mapped correctly onto the stimuli and our areas of interest on the left or the right side of the screen. The following numbers represent the participant that could not be calibrated across the groups: nine FXS, five DS, three PRE, and zero TYP. These numbers fall within the average range in our lab's experience for these populations and these ages [13-16].

**Figure 1:**
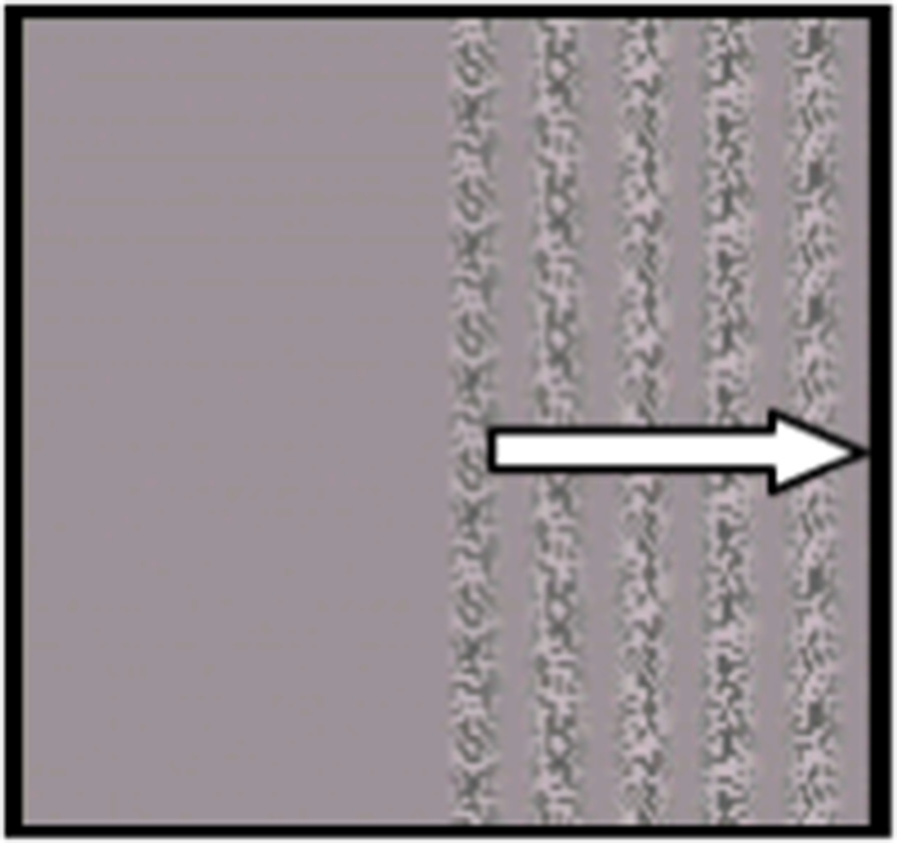
**Schematic example of visual stimuli used.** Second-order, texture-defined moving gradients. *Arrow* indicates direction of motion. Example shown is at a contrast level of 42%.

A forced-choice preferential looking procedure was utilized [34] in which stimuli were presented either on the left or the right side of the screen (see Figure 1) Each trial was approximately 3 s in length, with 1 s of the grating fading in (500 ms) and out (500 ms) of the screen. An attention getter was presented between trials to draw the participants' attention to the middle of the screen. This attention getter was a colorful, centrally located circle looming in and out accompanied by a single 3-s tone. Trials began automatically after the presentation of the attention getter. The presentation included four contrast levels (10%, 21%, 31%, or 42%), with the lowest contrast being the most difficult to perceive and the highest contrast being the easiest to perceive. There were a total of 40 trials (10 at each contrast level). The side of the screen that presented the stimuli was counterbalanced, and the contrast level randomized, across trials.

Following the same procedure used in Farzin et al. [13] after data acquisition at a frame rate of 50 Hz in Tobii Studio, a video recording of the stimuli overlaid with the eye tracking gaze data was exported to AVI format at 30 frames per second and imported into Noldus Observer 5.0 software (Noldus, Wageningen, The Netherlands) for manual coding. The coding protocol tracked gaze location (left, right, away, and center) on each trial. Center was defined as a fixation that was 50% on the left and 50% on the right of the midline of the screen. Coders were blind to the group status of the participant, and the inter-rater reliability for manual coding in Noldus was 97% (40 items; α = 0.97). Correct and incorrect visual responses were computed per trial, and a visual preference (VP) score was calculated at each contrast level. Correct looking was defined as looking to the half of the screen with the textured-defined gradient while incorrect looking was defined as looking at the side of the screen with the equiluminant gray display. The visual preference score was defined as the total looking time to the stimuli (correct looking)/the total looking time (correct and incorrect looking).

Contrast detection threshold for each participant was defined by calculating the visual preference score at each Michelson contrast level (10%, 21%, 31%, and 42%) and identifying the level (1-4) at which the participant could detect the stimuli on the screen. Visual preference scores of 75% or higher were used as a benchmark to determine the individual stimulus detection threshold. This benchmark was used in order to replicate the original contrast detection paper [13] and previous research in the adult vision literature [34-36]. Seventeen infants (one TD, four premutation, four DS, and eight FXS) did not reach a minimum preference score of 75% even at the highest contrast level. For analytic purposes, these infants were assigned a score of “4” along with those who reached a visual preference score of 75% or higher on *only* the highest contrast level. Thus, a contrast detection level score of 4 was given to those infants who could reliably see the gradient stimuli only at the contrast level of 42% *or* (theoretically) *higher*. Two infants (1 TD and 1 premutation) were excluded from the analyses because their preference scores were less than 50% across *all* contrast levels.

## Results

**Figure 2:**
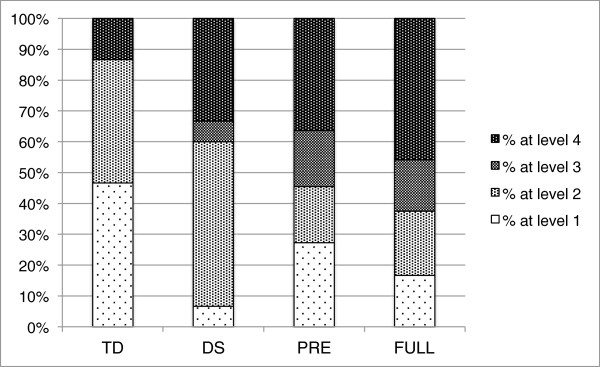
**Percentage of infants at each contrast detection threshold level**[1-3,13]**across the four participant groups.**

We carried out an ordered logistic regression to examine whether individuals in the various diagnostic groups have different probabilities of obtaining stimulus detection threshold levels at each of the four levels. In our analyses, diagnosis consisted of four groups (1 = TD; 2 = DS; 3 = FX premutation; and 4 = FXS) and contrast detection threshold level was an ordinal variable with four categories (1 = 10%; 2 = 21%; 3 = 31%; and 4 = 42% or higher), with percentages representing the amplitude of the second-order sinusoidal contrast modulation [13]. Our sample size consisted of *N* = 65 individuals. The distribution of individuals in each group as a function of the contrast detection threshold level is reported in Table 2 and depicted graphically in Figure 2.

**Table 2:** Number of infants at each contrast detection threshold across groups

**Contrast detection threshold level**	**Group**				**Total**
	**TD**	**DS**	**PRE**	**FXS**	
1	7	1	3	4	15
2	6	8	2	5	21
3	0	1	2	4	7
4	2	5	4	11	22
Total	15	15	11	24	65

The criteria used to determine a valid trial was based on fixations defined by the Clearview Fixation Filter (Tobii Technology; 35 velocity threshold and 100 duration threshold), defined by a minimum of a single fixation to either the left or the right side of the screen. The percentage of the total number of trials that were deemed valid across the groups (TYP, PRE, DS, and FXS) were 94%, 95%, 95%, and 90%, respectively. A 4 × 4 repeated measures ANOVA confirmed that there was no significant main effect of group *F*(3, 63) = 1.081, *p* = 0.364 and no significant group by threshold interaction *F*(3, 189) = 1.118, *p* = 0.343 interaction.

The various tests of the overall model were all significant, indicating that using diagnosis when predicting the probabilities of the contrasts was reasonable, as compared with a model without diagnosis (*χ*^2^[2] = 8.78, *p* = 0.03). The estimates for DS, FX premutation, or FXS were significant, denoting that belonging to any of these groups was associated with a lower probability of having a lower stimulus contrast detection threshold level, relative to the TD group, which served as the reference. For example, the estimate for DS was (*β* = -1.499, *p* = 0.031) indicating that, relative to the TD group, individuals in the DS group have a decrease of 1.5 in the odds of being in a lower level of contrast. The estimates for the premutation and FXS groups showed the same pattern (*β* = -1.417, *p* = 0.058; and *β* = -1.89, *p* = 0.003, respectively).

**Figure 3:**
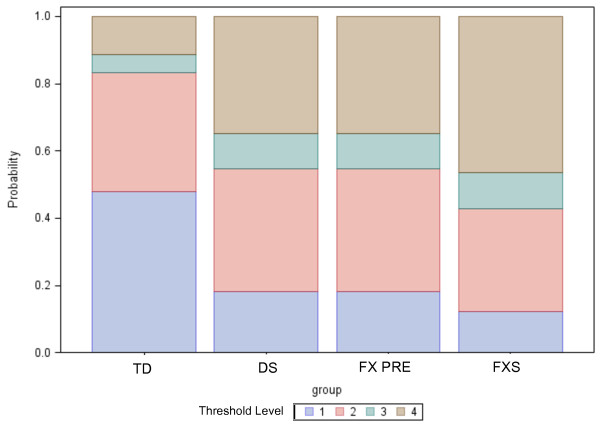
**Predicted probabilities for contrast detection threshold level.** Predicted probabilities for contrast detection threshold level [1-3,13] in the four participant groups.

The proportional odds ratios (coefficients exponentiated) show the assignment of contrast detection threshold level across the groups in terms of odds. For example, the odds ratio of TD vs. DS is 4.476, suggesting that individuals in the TD group are 4.5 times more likely to obtain a lower contrast detection threshold level than those in the DS group. The odds ratio comparing TD with FX premutation and with FXS denote that individuals in the TD groups are 4.1 and 6.6 times more likely to obtain a lower contrast detection threshold level than individuals in the FX premutation group or the FXS group, respectively. The odds ratios for these comparisons are displayed in Table 3, including the 95% confidence limits of the odds ratios. Figure 3 represents the predicted probabilities associated with scoring on each category of the contrast across the four groups.

**Table 3:** Confidence interval for odds ratio for each group comparison

	**Wald confidence interval for odds ratios**		
	**Estimate**	**95% confidence limits**	
Group 1 vs 2	4.476	1.145	17.500
Group 1 vs 3	4.115	0.948	17.852
Group 1 vs 4	6.621	1.866	23.498
Group 2 vs 3	0.919	0.223	3.785
Group 2 vs 4	1.479	0.453	4.834
Group 3 vs 4	1.609	0.435	5.949

Group 1 = TD, group 2 = DS, group 3 = FX premutation, group 4 = FXS.

## Discussion

The primary aim of this study was to provide a better understanding of the low-level visual processing mechanisms present in infant and toddler carriers of the FX premutation. Using a stimulus detection task for which we had previously demonstrated visual impairment (reduced contrast sensitivity) in infants with FXS [13], we hypothesized that although the phenotypic outcome of FX premutation carriers is much more favorable than those with FXS, this deficit in low-level visual processing would nonetheless be present. Our results confirmed that FX premutation carriers shared the same likelihood as full mutation carriers of having a contrast detection threshold level that was significantly higher (indicating poorer performance) than typically developing infants matched on mental age. We also tested a comparison group of infants with DS who were both chronologically and mentally age-matched to the participants with FXS and mentally age-matched to the infants with the FX premutation, and found that their performance did not differ significantly from the two FX groups. This result is consistent with research showing that individuals with DS exhibit significantly reduced visual acuity and contrast sensitivity as compared to TD controls [37] and suggests that this deficit may not be specific to the FX spectrum but a more general deficit found in other developmental disabilities such as Williams syndrome and autism spectrum disorders [30,38].

While it was long believed that individuals with the FX premutation remained cognitively unaffected throughout adulthood, there have been a number of studies, particularly those utilizing brain imaging techniques, which have documented measurable differences in brain functioning, across a number of different cognitive domains, in young adult premutation carriers who are asymptomatic for FXTAS [7,8,26]. Adult male FX premutation carriers have also been shown to exhibit slower reaction times, even after controlling for simple reaction time, in the visuospatial tasks of magnitude comparison and enumeration [28]. Perhaps most relevant to the present study, a recent investigation has shown that adult female FX premutation carriers show a 'dorsal stream deficit’ in that they are selectively impaired on perceptual tests of magnocellular (so-called M pathway—projecting primarily to dorsal visual stream areas) stimuli, whereas they show intact performance on tests of parvocellular ('P pathway’—projecting primarily to ventral visual stream areas) stimuli [29].

The present study is unique in that it is the first to document dorsal stream processing difficulties in infants and toddlers with the FX premutation. Our results suggest that even in very young premutation carriers, who in the vast majority of cases are cognitively unaffected and developing normally, a selective visual motion processing impairment on second-order motion gradient stimuli is present that is not significantly different from that found in those with the FX full mutation. While these results are striking, they beg the important question of what is the functional importance of such an impairment. It may be, as suggested by Keri and Benedek [29], that this represents a psychophysical endophenotype (a marker of genetic traits that do not result in observable clinical symptoms) for the FX spectrum of involvement. If so, individual differences in this ability may hold clues for differentiating those individuals on the FX spectrum (which includes premutation carriers, mosaics, and full mutation individuals) who are at risk for developing more severe phenotypes.

As reviewed above, there have been numerous demonstrations of spatiotemporal deficits in both individuals with FXS and those with the FX premutation. Because representations of space and time are integral to forming conceptions of number and arithmetic [31], impaired spatiotemporal processing may in fact also underlie the impairments in numerical processing that have been observed in both FXS [21] and in FX premutation carriers [26,39]. In females with FXS, brain activation during arithmetic processing has also been shown to be related to FMRP expression [21], which suggests that impaired processing of spatiotemporal information that is mediated primarily by the parietal cortex may represent an endophenotype that is modulated by *FMR1* gene expression across the FX spectrum. While the present study was underpowered to study how second-order motion visual processing may be modulated by variations in *FMR1* gene expression, this is an important question for future research to address.

Despite the spatiotemporal processing impairments in individuals with the FX premutation demonstrated both in the present study and elsewhere [28,29], the fact remains that individuals with the FX premutation only rarely present with overall cognitive functioning that falls below the normal range. One might be tempted, then, to dismiss these low-level visual processing impairments as unimportant to the individual’s perceptual and cognitive development. However, the presence of such impairments, even in an individual whose overall cognitive development falls within the normal range, may force compensation in the developing neural system, thus changing the landscape of development in ways that are difficult to measure, though potentially still impactful. It is therefore imperative that we continue to study and broaden our understanding of the processing capabilities of individuals with the FX premutation, particularly early in life.

## Conclusions

We tested four groups of infants and toddlers (TD, DS, FX premutation, and FXS) on a second-order motion stimulus detection task for which we had previously demonstrated visual impairment (reduced contrast sensitivity) in infants with FXS [13]. As reported previously, infants with FXS showed motion contrast detection threshold levels that were significantly higher than age-matched typically developing control infants. Strikingly, the motion detection contrast levels of FX premutation infants were also significantly higher than TD infants, and not significantly different from the group of infants with FXS or with the DS group. The present data, along with other evidence of impairments in processing spatiotemporal information that comes from the study of adults with the FX premutation, suggest that this type of spatiotemporal processing impairment may constitute an endophenotype for individuals on the FX spectrum and highlights the need for further study of the development of these processes, especially in children with the FX premutation.

## Abbreviations

FX: fragile X; FXS: fragile X syndrome; FMRP: fragile X mental retardation protein; CGG: cytosine-guanine-guanine; DS: Down syndrome; FXTAS: fragile X tremor/ataxia syndrome; VP: visual preference; TD: typically developing.

## Competing interests

The authors declare that they have no competing interests.

## Authors’ contributions

PG collected and analyzed the eye tracking data and wrote the first draft of the manuscript. JB assisted in the collection and analysis of the eye tracking data and provided feedback on the drafts of the manuscript. SR conceptualized and designed the study and wrote the final draft of the manuscript. All authors read and approved the final manuscript.

## Authors’ information

PG has a Masters in Psychology and is a research staff member (assistant specialist) in SR's Neurocognitive Development lab. JB has a BS in Psychology and is currently a graduate student in Psychology at UC Davis. SR has a Ph.D. in Psychology and is currently a Professor in the Department of Psychology at UC Davis.
